# Pleomorphic adenoma of the tonsillar cavity

**DOI:** 10.1016/j.ijscr.2024.109950

**Published:** 2024-06-26

**Authors:** Firas Maalej, Amal Samet, Manel Bouhali, Imene Rejab

**Affiliations:** a Department of ENT, University Hospital Habib Bourguiba of Sfax, Faculty of Medicine of Sfax, University of Sfax, Tunisia; b Department of Emergency, University Hospital of Gabes, Faculty of Medicine of Sfax, University of Sfax, Tunisia; c Department of Antomopathology, University Hospital of Gabes, Faculty of Medicine of Sfax, University of Sfax, Tunisia

**Keywords:** Pleomorphic adenoma, Palatine tonsil, Surgery

## Abstract

**Introduction and importance:**

The tonsillar location of pleomorphic adenomas is rare in histological diagnosis. The elimination of other essentially lymphomatous diagnoses is essential.

**Case presentation:**

We present a case of a 15-year-old child who consults for a feeling of pharyngeal discomfort and difficulty eating solid foods for 6 months. Clinical examination and radiology (MRI) showed the presence of a mass in the tonsillar region. A biopsy revealed a pleomorphic adenoma. The tumor was removed transorally with good progress.

**Clinical discussion:**

Pleomorphic adenoma of the tonsillar region is rare. Only histological examination can confirm this. Resection of the tumor must be complete in order to limit the risk of recurrence.

**Conclusion:**

The pleomorphic adenoma of the tonsillar region has a non-specific clinical presentation. MRI helps guide the diagnosis. Its treatment is surgical requiring complete excision.

## Highlights

• Plemorphic adenoma of the tonsillar compartment is rare
• Its clinical presentation is atypical.
• MRI imaging guides the diagnosis.
• The diagnosis is histological.
• Surgery must be complete to prevent recurrence.

## 1 Introduction

Salivary gland tumors affect the parotid gland in 70 % of cases, other less common locations such as the submandibular gland and the palate (in 8.4 and 8 % respectively). The tonsillar location of tumors remains exceptional, it represents only 0.5 % of locations [1].

Pleomorphic adenoma (PA) is the most common benign salivary gland tumor (65 to 75 %) [2]. It often affects adulthood with an average age of 47 years [3], children are rarely incriminated. In addition to age, risk factors may be related to smoking habits, alcohol abuse, a diet high in cholesterol and previous radiotherapy treatments in the cervico-facial straits. [2].

The aim of this article is to describe a case of PA of the minor salivary gland of the tonsillar compartment which is a rare entity with very few reported cases.

This article is written according to the 2023 Surgical CASe REport (SCARE) guideline [4].

## 2 Observation

This is a 15-year-old child with no specific pathological history, consulting the emergency room for a feeling of pharyngeal discomfort and difficulty eating solids for 6 months which has worsened in the last 24 h where the patient becomes aphagic. This symptomatology evolves without fever, odynophagia, dyspnea or dysphonia.

**Fig. 1:**
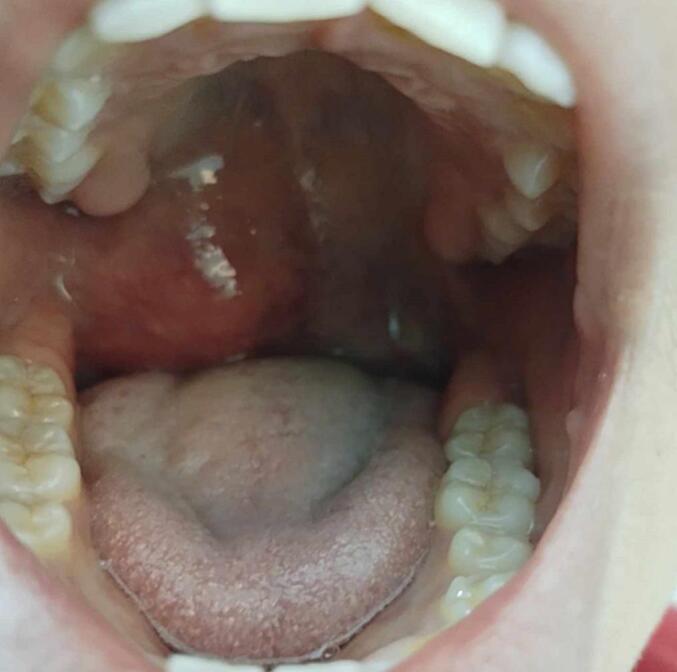
Bulging of the anterior pillar.

On examination, the patient was afebrile, eupneic; he presented a non-painful bulging of the right anterior pillar pushing the palatine tonsil downwards and backwards. The palatal tonsils were non-inflammatory and non-cryptic and the oropharynx was obstructive. (Fig. 1).

**Fig. 2:**
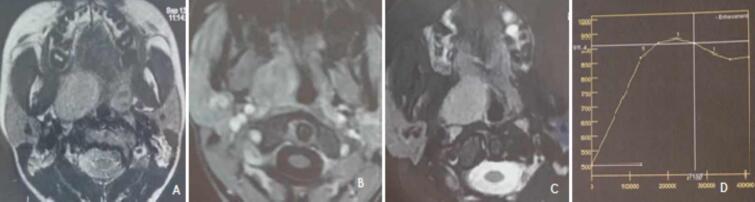
MRI of the oropharynx in axial section; A: T1 signal not injected; B: T1 signal injected; C: T2 signal; D: enhancement curve.

We completed with an MRI of the oral cavity which showed the presence of a 4 × 3 cm mass occupying the right tonsillar compartment in close relation with the right palatine tonsil, well limited without sign of loco-regional invasion, this mass was in signal intermediate T1, in T2 hypersignal and enhancing intensely and homogeneously with the contrast product. The enhancement curve was type A (Fig. 2).

**Fig. 3:**
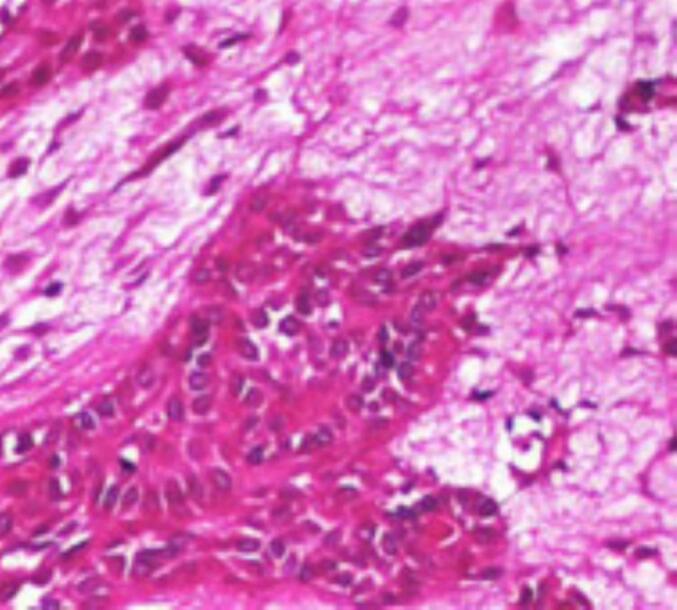
Pleomorphic adenoma: myoepithelial cells, epithelial cells and glandular elements dispersed in a myxomatous stroma.

A biopsy under local anesthesia was performed, concluding in a pleomorphic adenoma of the accessory salivary glands by the presence of myoepithelial cells, epithelial cells and glandular elements dispersed in the myxomatous stroma (Fig. 3).

The patient underwent intraoral surgery; we performed a single-piece excision of the mass with a right tonsillectomy. Antibiotic coverage with amoxicillin clavulanic acid was used. Histopathological examination of the surgical specimen confirmed the diagnosis of pleomorphic adenoma with healthy excision limits. The postoperative course was marked by the appearance of fluid reflux in the nasal cavity during feeding, which disappeared after 5 months. The follow-up period was one year without tumor recurrence.

## 3 Discussion

Pleomorphic adenoma (PA) is the most common benign tumor, accounting for 40–70 % of all tumors [2]. The palate is the most common site among the PA of the minor salivary glands. However, they can also occur in the larynx, trachea, floor of the mouth, upper lip and cheek [5]. Localization at the palatine tonsil is exceptional (0.5 %). Only a few cases have been published in the literature [[6], [7], [8], [9]].

The occurrence of AP can affect any age group, but it is particularly common in the 4th to 6th decades, with a predominance in women [1,2]. Although PA is the most common salivary tumor in children [10], tonsillar localization has not been described in the pediatric population in the literature.

The clinical presentation of pleomorphic adenoma of the oral cavity or oropharynx is nonspecific and included dysphagia, a feeling of blocked ears, nasal obstruction and recurrent sore throat, a feeling of choking or apnea of the summit [7,9], rarely trismus, cervical mass or cranial nerve deficit [11].

Imaging (Computed Tomography: CT, Magnetic Resonance Imaging: MRI) of the neck are essential diagnostic tools that allow the operating surgeon to determine the extent of the lesion, the type of tumor and the plan of approach. It has an important place for tumors with parapharyngeal extension. We note a superiority of MRI compared to CT, in terms of sensitivity, specificity of the detection of parapharyngeal AP and its locoregional extension [12]. Lesion localization is aided by the movement patterns of fat and the internal carotid artery in the parapharyngeal space [13]. The tumor appears in T2 hypersignal and in diffusion sequence. The T1 sequence signal varies depending on the degree of cystic or hemorrhagic remodeling. The apparent diffusion coefficient of the tumor is >1.7 · 10^−3^ mm^2^/s and type A contrast enhancement curve, the scanner being more efficient than MRI for the evaluation of bone relationships and a good alternative if MRI is not performed [12].

The preferred treatment is surgical excision. [14,15] The transoral route is ideal for palatine tonsil tumors. Depending on the extent of the tumor, the procedure can be reduced to a simple tonsillectomy. It is possible to extend to the anterior or posterior pillar for larger tumors. In parapharyngeal forms, excision is carried out via the submandibular transcervical route associated with the transoral route [16]. It happens that the capsule is pierced by microscopic extensions similar to pseudopods. Therefore, capsule rupture, inadequate resection, or tumor spillover at the time of excision may lead to local recurrence. [17] Approximately 6 % of patients are reported to experience recurrence. Late recurrences and malignant transformations have been reported, making long-term follow-up mandatory [18]. The risk of infection at the surgical site is greater than that of the trans-cervical route, because the excision is carried out in the septic environment [16] Antibiotic treatment is then recommended.

Histologically, the tumor consists of mesenchymal, epithelial, and myoepithelial arranged in a complicated manner. Epithelial cells secrete glandular and ductal substances containing eosinophil. Keratin pearls as well as squamous metaplasia are also present. The particularities of AP are the myoepithelial cells: clear, spindle-shaped and also often oxyphilic. The mesenchymal element of the tissue forms the chondroid, myxoid and bony zones. The false capsule usually occurs after fibrosis of the salivary gland parenchyma [19].

Myoepithelioma is the main histopathological diagnosis distinct from PA. The absence or paucity of ductal formations is the main difference between the presence of myoepithelial cells throughout the extension of myoepithelioma. In PA, most myoepithelial cells are present in variable numbers and ductal formations are present in large numbers [20].

The prognosis of PAs in the amygdalar compartment is generally good. However, the risk of recurrence is greater in extensive forms with incompletely resected pseudopods or with capsular rupture [16]. The prognosis also depends on the presence of malignant transformation; this risk can reach 3 to 14 % of AP [21].

## 4 Conclusion

Tumors of the accessory salivary glands at the level of the tonsillar compartment are rare. The symptomatology is atypical, often obstructive in the aero-digestive tract. A biopsy is necessary to rule out lymphoma. The treatment is surgical, the approach of which depends on the extent of the tumor. The main risks are recurrence and degeneration.

## Consent

Written informed consent was obtained from the patient's parents/legal guardian for publication and any accompanying images. A copy of the written consent is available for review by the Editor-in-Chief of this journal on request.

## Trial registration number/date

This is not a therapeutic trial.

## Ethical approval

This study is exempt from ethnic approval in our institution because it is a retrospective study whose surgical treatment is validated by learned societies without testing a new technique or new drug.

## Funding

N/A.

## Author contribution

Firas Maalej: data collection, surgery and writing of the article.

Amal Samet: collection of data in the emergency room and verification of writing in English.

Manel Bouhali: data collection, histopathological examination.

Imen Rejab: collection of data in the emergency room.

## Guarantor

Firas Maalej.

## Research registration number

This study is not the first in humans, it does not require registration.

## Conflict of interest statement

N/A.

## Data Availability

I declare that all the data in the medical file are available.
